# Global Significant Changes in Formaldehyde (HCHO) Columns Observed From Space at the Early Stage of the COVID‐19 Pandemic

**DOI:** 10.1029/2020GL091265

**Published:** 2021-02-23

**Authors:** Wenfu Sun, Lei Zhu, Isabelle De Smedt, Bin Bai, Dongchuan Pu, Yuyang Chen, Lei Shu, Dakang Wang, Tzung‐May Fu, Xiaofei Wang, Xin Yang

**Affiliations:** ^1^ School of Environmental Science and Engineering Southern University of Science and Technology Shenzhen China; ^2^ Division of Atmospheric Composition Royal Belgian Institute for Space Aeronomy (BIRA‐IASB) Brussels Belgium; ^3^ Department of Environmental Science and Engineering Shanghai Key Laboratory of Atmospheric Particle Pollution and Prevention Fudan University Shanghai China

**Keywords:** COVID‐19, HCHO, NMVOCs, TROPOMI

## Abstract

Satellite HCHO data are widely used as a reliable proxy of non‐methane volatile organic compounds (NMVOCs) to constrain underlying emissions and chemistry. Here, we examine global significant changes in HCHO columns at the early stage of the COVID‐19 pandemic (January–April 2020) compared with the same period in 2019 with observations from the TROPOspheric Monitoring Instrument (TROPOMI). HCHO columns decline (11.0%) in the Northern China Plain (NCP) because of a combination of meteorological impacts, lower HCHO yields as NO_*x*_ emission plunges (by 36.0%), and reduced NMVOC emissions (by 15.0%) resulting from the lockdown. HCHO columns change near Beijing (+8.4%) due mainly to elevated hydroxyl radical as NO_*x*_ emission decreases in a NO_*x*_‐saturated regime. HCHO columns change in Australia (+17.5%), Northeastern Myanmar of Southeast Asia (+14.9%), Central Africa (+7.8%), and Central America (+18.9%), consistent with fire activities. Our work also points to other changes related to temperature and meteorological variations.

## Key Points

- We detect significant changes in HCHO columns worldwide at the early stage of the COVID‐19 pandemic
- We see evidence of changing atmospheric oxidizing capacity and NMVOC emissions in the Northern China Plain due to the massive lockdown
- Besides anthropogenic emission, temperature and open fires are vital factors dominating variations in HCHO columns

## 1 Introduction

The COVID‐19 pandemic outbreak has reshaped normal social and economic activities dramatically, resulting in sudden changes in the emissions of air pollutants and their precursors. Recent air quality‐related studies predominantly focus on the impacts of plunged nitrogen oxides (NO_*x*_) emission (Bauwens et al., 2020; Liu et al., 2020; Menut et al., 2020; Venter et al., 2020; Zangari et al., 2020; R. Zhang et al., 2020) on particulate matters (Chang et al., 2020; G. He et al., 2020; Kumar et al., 2020; Shi & Brasseur, 2020; Venter et al., 2020; Zangari et al., 2020) and surface ozone (G. He et al., 2020; Le et al., 2020; Shi & Brasseur, 2020; Siciliano et al., 2020; Venter et al., 2020). Here, we use formaldehyde (HCHO) columns from the TROPOspheric Monitoring Instrument (TROPOMI) (Veefkind et al., 2012) to examine global changes in HCHO at the early stage of the COVID‐19 pandemic (hereafter defined as January–April 2020) and relate them to variations in anthropogenic emissions of non‐methane volatile organic compounds (NMVOCs), temperature, and open fires.

HCHO is detectable from space as a vertical column density (VCD) using solar ultraviolet backscattered radiation between 325 and 360 nm (Chance et al., 2000). Owing to its short atmospheric lifetime (a few hours against oxidation and photolysis) and high production yields from the oxidation of NMVOCs, HCHO VCD has been applied as a localized proxy for NMVOC emissions from biogenic sources (Palmer et al., 2003; Shim et al., 2005; Surl et al., 2018; Y. Zhang et al., 2019), anthropogenic sources (Fu et al., 2007; Shen et al., 2019; L. Zhu et al., 2014), and open fires (Gonzi et al., 2011; Shim et al., 2005; Y. Zhang et al., 2019).

In this study, we use HCHO data available from the recently launched TROPOMI to examine how HCHO changes from January–April 2019 to January–April 2020, building on our oversampling method (Sun et al., 2018; L. Zhu et al., 2014; L. Zhu, Jacob, et al., 2017), and identifying significance through allocating satellite pixels by their corresponding temperatures (L. Zhu, Mickley, et al., 2017)

## 2 Significant Changes in TROPOMI HCHO Columns

TROPOMI is a nadir‐viewing hyperspectral spectrometer onboard the Copernicus Sentinel‐5 Precursor platform launched in October 2017. It scans the whole globe daily at a local time of 13:30. We use the TROPOMI HCHO product (De Smedt et al., 2018) based on the technical heritage of retrieving HCHO from the Global Ozone Monitoring Experiment (GOME), GOME‐2, and Ozone Monitoring Instrument (OMI) (De Smedt et al., 2018). TROPOMI HCHO product offers the finest nadir spatial resolution (7 km × 3.5 km, upgraded to 5.5 km × 3.5 km since August 2019) with a high signal‐to‐noise ratio among currently available HCHO products. The product correlates highly with Multi‐AXis Differential Optical Absorption Spectroscopy (*r* = 0.88) and Fourier‐transform infrared (*r* = 0.91) measurements with a mean bias ranging from −26.0% to +30.8%, depending on locations (Chan et al., 2020; Vigouroux et al., 2020).

We select TROPOMI level‐2 pixels with (1) quality assurance value greater than 0.5, (2) cloud fraction less than 0.3, and (3) solar zenith angle less than 60°. This study focuses on examining significant mean changes in HCHO columns from January–April 2019 to January–April 2020, rather than investigating daily or weekly variations. We follow L. Zhu, Mickley, et al. (2017) to group TROPOMI pixels by their associated temperatures to identify significant changes between the two periods. Unlike focusing on a long‐term HCHO trend in L. Zhu, Mickley, et al. (2017), those changes between two sets of 4‐month observations are still influenced by monotonous temperature changes.

Briefly, we first assign a temperature value to each level‐2 pixel based on its location and observing time, using hourly surface air temperature data (0.5° × 0.625°) from the Modern‐Era Retrospective Analysis for Research and Applications, version 2 (MERRA‐2) (Gelaro et al., 2017). We then allocate all pixels into 200 temperature bins ranging from 273 to 323 K with an increment of 0.25 K. For each bin *i*, we use our oversampling method (Sun et al., 2018; L. Zhu et al., 2014; L. Zhu, Jacob, et al., 2017) to map mean January–April HCHO columns in 2019 and 2020 onto 0.5° × 0.5° grids (*j*), denoted as ${\bar{\Omega }}_{i,j,2019}$ and ${\bar{\Omega }}_{i,j,2020}$, respectively. We determine this spatial resolution based on a satellite detection capability and overlapping pixels amount over a grid cell. The change in HCHO columns in temperature *i* for grid cell *j* is written as

(1) $$\Delta {\bar{\Omega }}_{i,j}={\bar{\Omega }}_{i,j,2020}-{\bar{\Omega }}_{i,j,2019}$$

We further compute the change for each grid cell *j* ($\Delta {\bar{\Omega }}_{j}$) as the mean of changes across all bins with at least 30 overlapping pixels, weighted by the total number of overlapping pixels in each bin:

(2) $$\Delta {\bar{\Omega }}_{j}=\frac{\sum_{i}\Delta {\bar{\Omega }}_{i,j}\left(N_{i,j,2019}+N_{i,j,2020}\right)}{\sum_{i}N_{i,j,2019}+N_{i,j,2020}}$$

where *N*_*i,j*,2019_ and *N*_*i,j*,2020_ represent the number of overlapping pixels for grid cell *j* in temperature bin *i* for January–April of 2019 and 2020. We consider only grid cells with values from at least 50 temperature bins so that each $\Delta {\bar{\Omega }}_{j}$ is contributed by more than 1,500 pixels. We restrict our following analysis to grid cells with significant nonzero $\Delta {\bar{\Omega }}_{j}$, determined with the *t* test (*p*‐value < 0.05). Here and elsewhere, changes in TROPOMI HCHO columns are significant, unless otherwise stated. We also try a finer spatial resolution of 0.05° × 0.05° and 0.25° × 0.25° but find that it becomes difficult to obtain statistically significant outcomes from smaller sample sizes.

## 3 Results and Discussions

**Figure 1:**
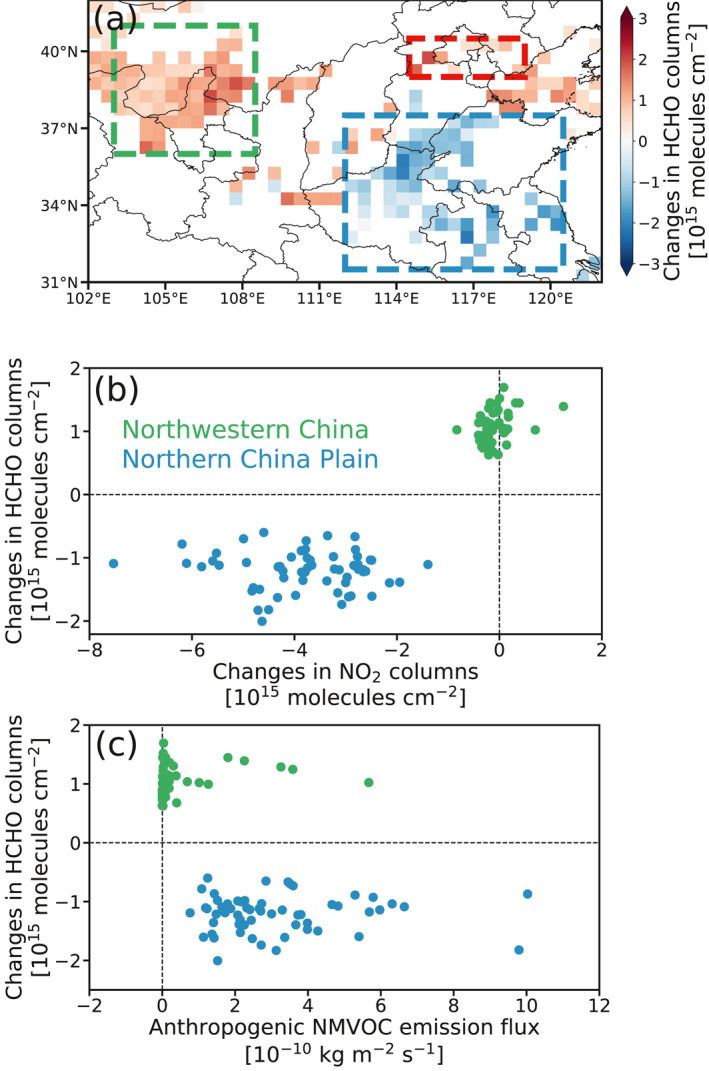
Significant changes in mean TROPOMI HCHO columns at the early stage of the pandemic (defined as January–April 2020) in (a) Northern China and its respective relations to (b) changes in TROPOMI NO_2_ columns and (c) anthropogenic NMVOC emissions. Changes are computed as the difference in mean columns from January–April 2019 to January–April 2020. The blue and green dots in (b) and (c) represent individual 0.5° × 0.5° grid cells with HCHO changes in the Northern China Plain (NCP; the blue box in (a); 112°E−120.5°E, 31.5°N–37.5°N) and Northwestern China (the green box in (a); 103°E−108.5°E, 36°N–41°N), respectively. The red box (114.5°E−119°E, 39°N–40.5°N) contains several grid cells near Beijing. TROPOMI NO_2_ pixels (van Geffen et al., 2020) are selected using the same criteria as HCHO pixels (see text) and are oversampled (without being grouped by temperatures) onto the 0.5° × 0.5° grids for the respective two periods. Anthropogenic NMVOC emissions are from the mosaic Asian anthropogenic emission inventories (MIX) (Li et al., 2017). TROPOMI, TROPOspheric Monitoring Instrument; NMVOC, non‐methane volatile organic compound.

As shown in Figure 1(a), HCHO columns decline on average by 11.0% ± 2.8% in the Northern China Plain (NCP) at the early stage of the COVID‐19 pandemic compared with the same period in 2019. Such a decrease is not driven by temperature (+0.7 K; Table S1) and not fully due to meteorological variations which only result in a 5.7% decrease in HCHO columns (Table S1). HCHO reductions are generally located at grid cells with predominant declines in NO_2_ columns (Figure 1(b)) and relatively high anthropogenic NMVOC emissions (Figure 1(c)), suggesting a linkage between reductions in HCHO columns and perturbations of anthropogenic emissions resulting from the massive lockdown. However, such a linkage is likely complicated by (1) varying HCHO production yields resulted from different NO_*x*_ levels (Wolfe et al., 2016; L. Zhu, Mickley, et al., 2017) as Chinese NO_*x*_ emission plunges in this period (Bauwens et al., 2020; Feng et al., 2020; Liu et al., 2020; Miyazaki et al., 2020; Shi & Brasseur, 2020) and (2) changing anthropogenic NMVOC emissions in the NCP, a region characterized by intense industry activities

(Li et al., 2019).

To tease out contributions by the above two factors, we conduct a series of sensitivity simulations with the GEOS‐Chem (http://www.geos-chem.org, version 12) 3‐D chemical transport model. We scale down NO_*x*_ and NMVOC emissions to simultaneously reproduce the mean reduction in NO_2_ (42.2%) and HCHO (11.0%) column seen by TROPOMI in the NCP. GEOS‐Chem is reliable in modeling the observed relationship between HCHO columns and NMVOC emissions (Fu et al., 2007; Palmer et al., 2003; Shen et al., 2019; Surl et al., 2018; L. Zhu et al., 2016, 2020) under various NO_*x*_ conditions (Travis et al., 2016; L. Zhu, Mickley, et al., 2017). Here, we run the GEOS‐Chem model (2° × 2.5°) from January 2018 to April 2020, driven by the MERRA‐2 meteorological fields (Gelaro et al., 2017). The model uses biogenic VOC emissions from the MEGAN v2.1 (Guenther et al., 2012), open fire emissions from the fourth‐generation global fire emissions database (Giglio et al., 2013), and anthropogenic emissions from the MIX inventory (Li et al., 2017) over Asia. We sample GEOS‐Chem outputs according to the TROPOMI schedule and then grid the model results to the same 0.5° × 0.5° grids of satellite observations. We acknowledge the coarse model resolution, which may not lead to biases as small‐scale nonlinearities may have an insignificant impact on regional analysis (Yu et al., 2016).

**Figure 2:**
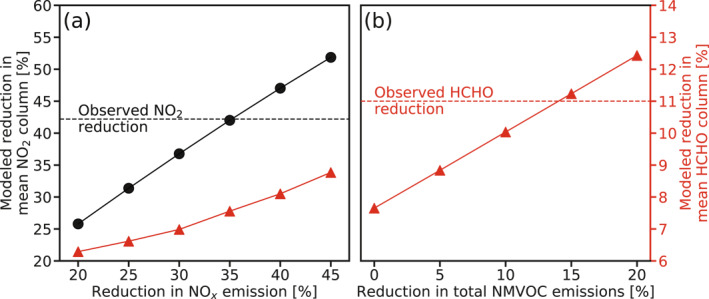
Responses of modeled reductions in mean NO_2_ and HCHO columns in the Northern China Plain (NCP) to the scaling‐down of NO_*x*_ and NMVOC emissions. (a) Reductions in mean NO_2_ (black dots) and HCHO columns (red triangles) due to declining NO_*x*_ emissions. The black dashed line demonstrates the decrease (42.2%) in mean NO_2_ columns from January–April 2019 to January–April 2020 in the NCP, as observed by TROPOMI. (b) Reductions in mean HCHO columns (red triangles) due to declining NMVOC emissions (with a fixed reduction of 36.0% in NO_*x*_ emission to match the observed decrease in TROPOMI NO_2_ columns). The red dashed line shows the reduction (11.0%) in mean HCHO columns according to TROPOMI. Model results are from a series of GEOS‐Chem sensitivity simulations run at 2.0° × 2.5°. NMVOC, non‐methane volatile organic compound; TROPOMI, TROPOspheric Monitoring Instrument.

Figure 2 summarizes how modeled mean NO_2_ and HCHO columns in the NCP respond to the scaling‐down of NO_*x*_ and NMVOC emissions. NO_2_ column reduction correlates almost linearly with the decrease in NO_*x*_ emissions on the regional scale. GEOS‐Chem reproduces the observed reduction in mean NO_2_ column (42.2%) in the NCP when applying a uniform decrease of 36.0% in anthropogenic NO_*x*_ emissions. The result takes the difference in meteorological conditions between 2019 and 2020 into consideration by running the GEOS‐Chem model with corresponding meteorological fields. Our finding is consistent with the recently estimated reductions in NO_2_ (40%–60%) and NO_*x*_ (36%–48%) in China during the COVID‐19 pandemic (Bauwens et al., 2020; Feng et al., 2020; Liu et al., 2020; Miyazaki et al., 2020; Shi & Brasseur, 2020).

We see from Figure 2(a) that the declining NO_*x*_ emission leads to additional lower HCHO columns in the NCP. This is due to the slower conversion of HO_2_ to OH caused by lower NO, which decelerates HCHO production from the oxidation of NMVOCs (Wolfe et al., 2016; L. Zhu, Jacob, et al., 2017; L. Zhu, Mickley, et al., 2017). Reducing NO_*x*_ emissions by 36.0% as constrained by TROPOMI NO_2_ observations results in only a 7.7% decline in the mean HCHO column in the NCP, inconsistent with the reduction according to TROPOMI (11.0%; Figure 1). This suggests that anthropogenic NMVOC emissions in the NCP probably also decrease at the early stage of the pandemic. Our simulations (Figure 2(b)) show that introducing an additional 15.0% reduction in anthropogenic NMVOC emissions is necessary to fill the gap, simultaneously reproducing observed mean reduction in both NO_2_ and HCHO columns in the NCP. Here, the scaling factors are applied to all emission sectors. We acknowledge that NO_*x*_ and NMVOC emissions from some sectors may have changed differently during the lockdown, and detailed atmospheric chemical relationship in urban scale deserves future exploration.

Figure 1(a) also shows that the mean HCHO column increases on average by 8.4% ± 4.2% over a few grid cells near Beijing. We attribute this to a faster HCHO production rate through the oxidation of NMVOCs, driven by higher OH levels as NO_*x*_ emission decreases (by 17.0%, according to TROPOMI NO_2_ columns) in a NO_*x*_‐saturated regime. GEOS‐Chem results confirm that the mean surface OH level increases by 14.5% ± 3.3% in this region during this period due both to a reduction in NO_*x*_ emissions (8.2%) and changes in meteorological conditions (6.3%).

The mean HCHO column raises by 14.2% ± 3.1% in Northwestern China (Figure 1(a)). This region is characterized by weak reductions in NO_2_ columns (Figure 1(b)) and low anthropogenic NMVOC emissions (Figure 1(c)), implying variations in meteorological conditions rather than anthropogenic emissions as the main driver. The surface temperature increases by 1.0 K on average, which translates to an increase of 11.1% in HCHO columns (Table S1), assuming an exponential dependence on temperature (Palmer et al., 2006; L. Zhu et al., 2014). Acknowledging such temperature dependence is from regional/local studies, we attribute HCHO changes here qualitatively to temperature variations. GEOS‐Chem fails to reproduce the HCHO increase in this region (Table S1), implying the impact of temperature has likely been over‐compensated by other factors in the model when HCHO is low.

**Figure 3:**
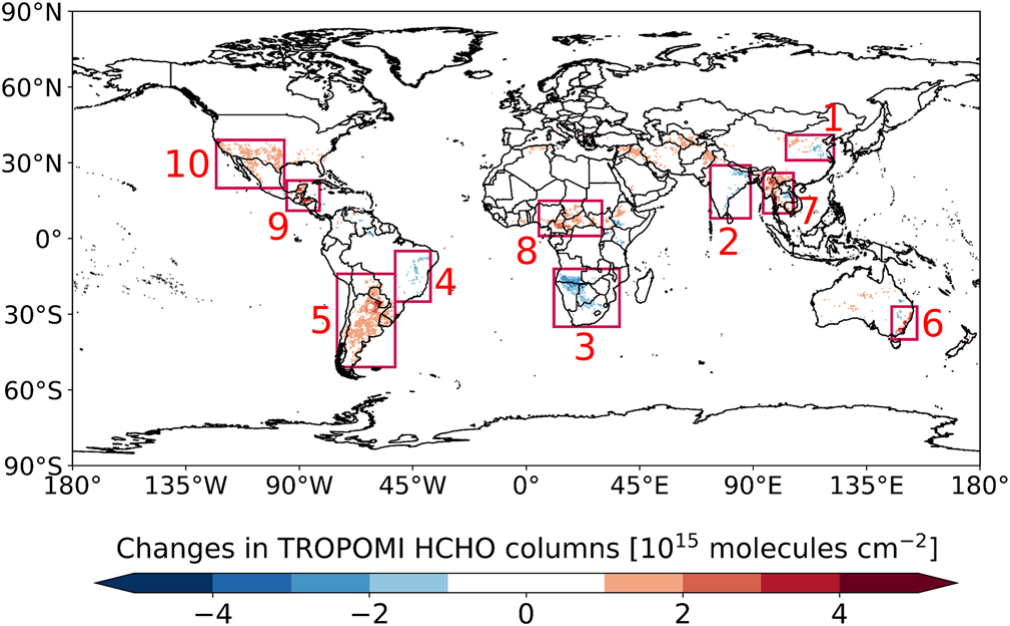
Global significant changes in HCHO columns (0.5° × 0.5°) at the early stage of the pandemic, computed as the difference in mean TROPOMI HCHO columns from January–April 2019 to January–April 2020. Changes in 10 regions are examined in the text and labeled in order 1–10: Northern China (same as the domain shown in Figure 1(a)), India, Southern Africa, Eastern Brazil, Southern Cone, Southeastern Australia, Southeast Asia, Central Africa, Central America, and the Southwestern United States and Northern Mexico. TROPOMI, TROPOspheric Monitoring Instrument.

In Figure 3, we extend our analysis to examine global changes in HCHO columns at the early stage of the pandemic. In India (Region 2), the mean HCHO column decreases by 7.0% ± 2.9% on a subcontinental scale, consistent with modeled reduction (6.3%) due to meteorological difference (Table S1). HCHO reduction is also roughly consistent with an estimated reduction (9.8%) when applying the temperature dependence (Table S1). Those findings likely reflect the dominant influence of temperature (−0.9 K) in biogenic isoprene emission, thus on HCHO columns in India (Surl et al., 2018). We observe similar temperature‐dominated influence on HCHO columns in Southern Africa (Region 3; −11.7% ± 6.4%), Eastern Brazil (Region 4; −10.1% ± 4.6%), Southern Cone (Region 5; +13.5% ± 5.1%), and Northeastern Thailand in Southeast Asia (Region 7; −11.2% ± 2.5%), where regional mean temperature changes respectively by −1.8, −1.0, +0.8, and −1.4 K, as summarized in Table S1.

The increase in HCHO columns in Southeastern Australia (Region 6; 17.5% ± 10.7%) may be traced to exceptionally high open fire emissions at the beginning of 2020, but not meteorological variations (Table S1). Using the Global Fire Assimilation System (GFAS) data (Kaiser et al., 2012), we find mean non‐methane hydrocarbon (NMHC) emission flux increases by a factor of ∼7.0 in this region. We see similar correlations between changes in HCHO columns and fire activities in Northeastern Myanmar in Southeast Asia (Region 7; +14.9% ± 3.4%), Central Africa (Region 8, +7.8% ± 3.7%), and Central America (Region 9; +18.9% ± 7.8%), where regional mean NMHC emission flux changes by +28.4%, +18.5%, and +19.5%. Those regions are known for high fire‐driven HCHO columns (Marbach et al., 2008; Stavrakou et al., 2015). However, quantifying the impact of fire emissions on HCHO columns is challenging because of the uncertainties in diurnal variations and emission factors of wildfires. In the Southwestern United States and Northern Mexico (Region 10), the mean HCHO column increases by 12.2% ± 0.1% with unclear reasons (Table S1).

The lack of significant anthropogenic signals outside the NCP at the early stage of the COVID‐19 pandemic (Figure 3) is consistent with the lockdown timeline worldwide. We acknowledge that the absence of TROPOMI HCHO observations before May 2018 limits the long‐term analysis of HCHO columns. Although OMI HCHO (González Abad et al., 2015) has been widely used for long‐term trend analysis (De Smedt et al., 2015; Shen et al., 2019; L. Zhu, Mickley, et al., 2017b; S. Zhu et al., 2018), we find OMI fails to detect significant HCHO changes (Figure S1 and Text S1). This is due to inadequate valid observations from OMI, thus emphasizing the value of TROPOMI data. Future studies to investigate interannual and urban‐scale variabilities in HCHO columns and NMVOC emissions may become available as a longer span of TROPOMI HCHO product or Geostationary HCHO observations (Kim et al., 2020; Kwon et al., 2019) are ready.

## 4 Conclusion

We have used TROPOMI satellite observations to examine significant changes in HCHO columns at the early stage of the COVID‐19 pandemic over the globe. We find regional decline driven by reduced anthropogenic nitrogen oxides (NO_*x*_) and NMVOC emissions in the NCP. Regional changes in Northwestern China, India, Southern Africa, Eastern Brazil, Southern Cone, and Northeastern Thailand may be traced to variations in temperatures. The impact of open fires on HCHO columns is also identified in Southeastern Australia, Northeastern Myanmar, Central Africa, and Central America. Our study highlights the importance of TROPOMI satellite observations in understanding variations in anthropogenic emissions by providing evidence from space.

## Supporting information

Supporting Information S1

## Data Availability

The GEOS‐Chem 3‐D Chemical Transport Model is available at http://www.geos-chem.org.
