# Supplementary material for: Global Significant Changes in Formaldehyde (HCHO) Columns Observed From Space at the Early Stage of the COVID‐19 Pandemic
Source: Geophys Res Lett. 2021 Feb 23;48(4):2e020GL091265. doi: 10.1029/2020GL091265 (PMC7995117; doi:10.1029/2020GL091265)
Supplement: Supplementary file 1 — Supporting Information S1 [file GRL-48-2e020GL091265-s001.pdf]

Geophysical Research Letters

Supporting Information for

Global Significant Changes in Formaldehyde (HCHO) Columns Observed from Space at the Early Stage of the COVID-19 Pandemic

Wenfu Sun[1], Lei Zhu[1,*], Isabelle De Smedt[2], Bin Bai[1], Dongchuan Pu[1], Yuyang Chen[1], Lei Shu[1], Dakang Wang[1], Tzung-May Fu[1], Xiaofei Wang[3], and Xin Yang[1]

[1]School of Environmental Science and Engineering, Southern University of Science and Technology, Shenzhen, China.

[2]Belgian Institute for Space Aeronomy (BIRA-IASB), Brussels, Belgium.

[3]Shanghai Key Laboratory of Atmospheric Particle Pollution and Prevention, Department of Environmental Science and Engineering, Fudan University, Shanghai, China.

*** Corresponding author:**

* Lei Zhu, Email: zhul3@sustech.edu.cn

Contents of this file

Texts S1
Figures S1
Tables S1

Introduction

This Supporting Information provides 1 supplementary text, 1 supplementary figure, and 1 supplementary table to support the discussions in the main text. The contents of these supplementary materials are as follows.

Text S1. The processing of OMI HCHO products and the description of outputs.

Figure S1. Global changes in HCHO columns (0.5°×0.5°) at the early stage of the pandemic, computed as the difference in mean Ozone Monitoring Instrument (OMI) HCHO columns from January–April 2019 to January–April 2020.

Table S1. Changes in mean HCHO columns from January–April 2019 to January–April 2020 over different regions with significant changes.

Text S1.

The HCHO columns are from OMI HCHO level-2 products (Gonzalez Abad *et al.*, 2015), and we select OMI pixels with (1) good data quality (main data quality flag is 0), (2) cloud fraction less than 0.3, and (3) solar zenith angle less than 60°. Gonzalez Abad *et al.* (2015) corrected OMI HCHO products from signal drift with a reference sector re-normalization using a GEOS-Chem derived monthly climatology over the remote Pacific Ocean.

As shown in Figure S1, we fail to see a clear pattern of changes in mean OMI HCHO columns from January–April 2019 to January–April 2020, computed by direct subtraction, despite we lower the overlapping pixels amount requirement to a minimal 100. It is mainly due to insufficient effective pixels for two reasons: (1) the spatial resolution is coarse, and (2) the row anomaly makes it necessary to abandon 35 rows in a scan during the oversampling (60 rows in total for a scan) to avoid the strip effect. Moreover, we fail to test the statistical significance of OMI HCHO columns' changes as few pixels would be assigned into temperature bins over a grid.

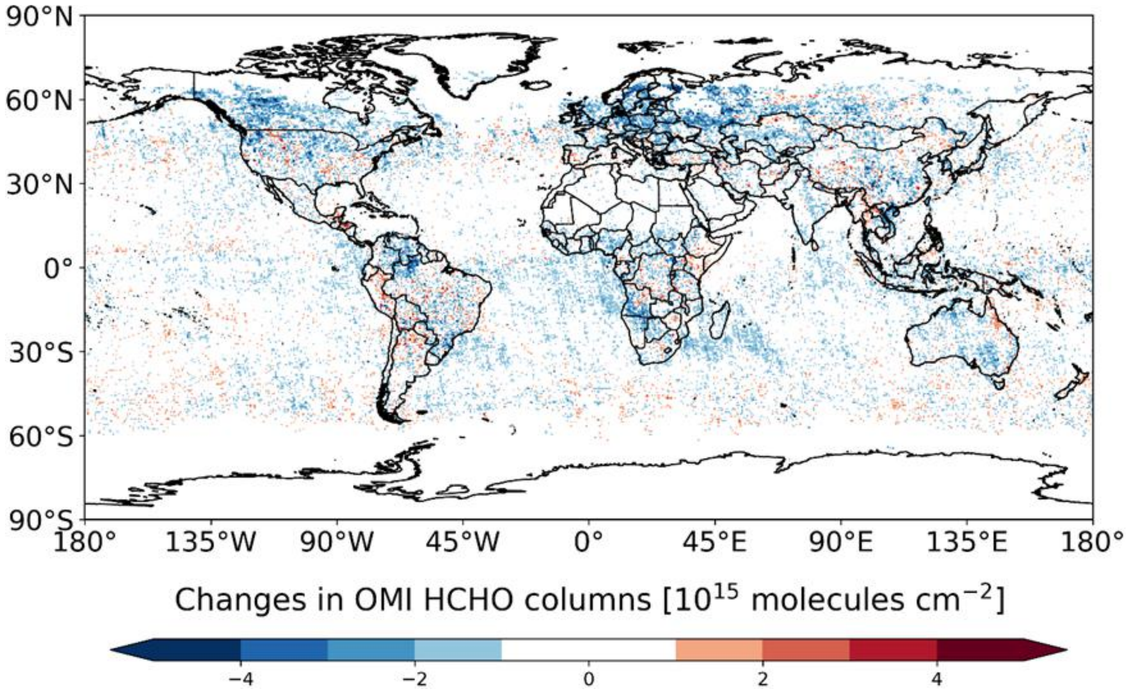

Figure S1. Global changes in HCHO columns (0.5°×0.5°) at the early stage of the pandemic, computed as the difference in mean Ozone Monitoring Instrument (OMI) HCHO columns from January–April 2019 to January–April 2020. The OMI HCHO level-2 pixels (Gonzalez Abad *et al.*, 2015) are selected with the criteria introduced in Text S1 and are oversampled (without being grouped by temperatures) onto the 0.5°×0.5° grids for the respective two periods.

Table S1. Changes in mean HCHO columns from January–April 2019 to January–April 2020 over different regions with significant changes.

Region		Observed change in mean HCHO columns	Change in Surface temperature (K)	Estimated change in mean HCHO columns [1]	Modeled change in mean HCHO columns [2]
1	Northern China:				
	Northern China Plain	−11.0%	+0.7	+8.1%	−5.7%
	Northwestern China	+14.2%	+1.0	+11.1%	−5.9%
2	India	−7.0%	−0.9	−9.8%	−6.3%
3	Southern Africa	−11.7%	−1.8	−17.7%	−15.0%
4	Eastern Brazil	−10.1%	−1.0	−10.7%	−6.5%
5	Southern Cone	+13.5%	+0.8	+9.2%	+8.4%
6	Southeastern Australia	+17.5%	−1.7	−17.4%	−20.2%
7	Southeast Asia:				
	Northeastern Thailand	−11.2%	−1.4	−14.2%	−11.4%
	Northeastern Myanmar	+14.9%	+0.02	+0.2%	−1.0%
8	Central Africa	+7.8%	−1.0	−10.2%	−9.3%
9	Central America	+18.9%	+0.7	+7.8%	+6.5%
10	Southwestern U.S. & Northern Mexico	+12.2%	+0.5	+6.0%	+0.5%

Note: [1] The change in mean HCHO columns is estimated with the exponential fit of HCHO columns (Ω) to the surface air temperature (T) as $\ln\Omega = 2.62 + 0.11T$ derived by Zhu *et al.* (2014). [2] Simulated by the GEOS-Chem model (see the main text) with fixed emissions but different meteorological fields for 2019 and 2020; here changes should purely reflect the impacts of meteorological variations.

References.

Gonzalez Abad, G., Liu, X., Chance, K., Wang, H., Kurosu, T. P., & Suleiman, R. (2015). Updated Smithsonian Astrophysical Observatory Ozone Monitoring Instrument (SAO OMI) formaldehyde retrieval. *Atmospheric Measurement Techniques, 8*(1), 19-32. https://doi.org/10.5194/amt-8-19-2015

Zhu, L., Jacob, D. J., Mickley, L. J., Marais, E. A., Cohan, D. S., Yoshida, Y., et al. (2014). Anthropogenic emissions of highly reactive volatile organic compounds in eastern Texas inferred from oversampling of satellite (OMI) measurements of HCHO columns. *Environmental Research Letters, 9*(11). https://doi.org/10.1088/1748-9326/9/11/114004
